# Supplementary material for: Hypothesis: Single Actomyosin Properties Account for Ensemble Behavior in Active Muscle Shortening and Isometric Contraction
Source: Int J Mol Sci. 2020 Nov 9;21(21):8399. doi: 10.3390/ijms21218399 (PMC7664901; doi:10.3390/ijms21218399)
Supplement: Supplementary file 1 [file ijms-21-08399-s001.pdf]

Supporting Information

Hypothesis: Single actomyosin properties account for ensemble behavior in active muscle shortening and isometric contraction

by
Alf Månsson[1]

[1] Linnaeus University, Dept. of Chemistry and Biomedical Sciences, Universitetskajen, 391 82 Kalmar.

Supporting Text

The parameter values used in the model simulations in the main paper are given in Tables S1 and S2 and the rate functions are given in Table S3. The latter functions are inserted into the ordinary differential equations used to obtain steady-state probability distributions vs the actin-myosin strain coordinate x for the different cross-bridge states[1, 2]. These probability distributions are then used to obtain steady-state force, stiffness, ATP turnover rates etc.[1, 3]. The rate functions in Table S3 with parameter values as in Tables S1 and S2 are also used to obtain stochastic waiting times between subsequent state transitions and stochastic selection of the actual transition for each event according to the Gillespie algorithm[4] (see further [5]), when simulating transient events and events involving few (<100) myosin molecules.

The origin of the parameter values are indicated by references in Tables S1 and S2 but are commented on in greater detail below. Importantly, all parameter values are obtained under as similar experimental conditions as possible using isolated myosin (full length, subfragment 1 or heavy meryomyosin) from fast skeletal muscle of the rabbit. Further, all values refer to ionic strengths of 100-200 mM, pH 7-8 and temperature of 30 ^{o}C unless otherwise stated in Tables S1-S2.

Parameter values 1: x-values for positions of free energy minima of different states

First, a coordinate system that describes the actomyosin distortion (strain) is defined, so that x=0 nm when the free energy of the rigor (AM) state is at its minimum value, thus fixing x_3 at x_3=0 nm. Moreover, the total power-stroke distance (x_{11} - x_3 = (x_{11} – x_2) + (x_2 - x_3); see main Fig. 3B), assumed here and in most other studies to involve more than one stroke[6], has been found to be in the range 7 – 13 nm[6] suggesting that x_{11} is in this range. In the simulations, I use a cross-bridge stiffness value (ks=2.8 pN/nm) that I judge to have credible support in single molecule mechanics measurements using full length myosin[7]. This implies a total power-stroke distance (x_{11}-x_3) close to the lower bound of the 7-13 nm range because the elastic energy ($ks(x_1-x_3)^2/2$) reaches the free energy of ATP turnover (25 k_BT) for x_1=8.4 nm. Together with a maximum thermodynamic efficiency of muscle of less than 50 %[8] it follows from this result that (x_{11}-x_3) is likely to be lower than 8.4 nm. In our initial modelling[3] we used x_{11}=8.2 nm, later optimized (within experimental uncertainties) in attempts to account for effects of varied concentrations of inorganic phosphate (Pi), to x_{11}=6.7 nm. It should be noted that the latter value relies on the assumption that cross-bridge stiffness is 2.8 pN/nm (see above). If a stiffness value of ~1.5 pN/nm is instead assumed, as suggested by some studies[9, 10], the quantity $ks(x_{11}-x_3)^2/2$) attains 25 k_BT for x_{11}=11.5 nm rather than for x_{11}=8.4 nm, consistent with the rather broad range of the power-stroke distance in the literature (reviewed in [6]). Now, there is ample evidence for more than one force-generating power-stroke. Whereas some authors have assumed two or more power-strokes of similar amplitudes (e.g. [11-13]), most studies (e.g. [14-16]; reviewed in [6]) suggest that a large major power-stroke (between the AMD_L and AMD_H states in present model; amplitude (x_{11}-x_2)) is followed by a second smaller stroke, presumably associated with strain-dependent release of ADP (between AMD_H and AMD/AM; amplitude x_2-x_3) (see further main Fig. 3). Single molecule mechanics data suggest that the amplitude x_2-x_3 is in the range 0.9 – 2.5 nm[14, 15]. In my initial modelling efforts (when the higher estimate of 2.5 nm[15] was not available) I used x_2=1 nm and now continue to do so. However, if a higher numerical value of x_2 is corroborated in future studies the model will be changed accordingly. Finally, the parameter value x_1 that gives the position of the free energy minimum of the pre-power-stroke state was introduced (in addition to the parameter represented by x_{11}) in efforts to model effects of the small molecular myosin inhibitor blebbistatin[3]. Initially we assumed that x_1=x_{11}+1nm but this was later[17] changed to x_1=x_{11}+0.5nm to accommodate effects of varied [Pi]. Such changes are within the experimental uncertainties because no real quantitative estimate exists for the difference x_1-x_{11}. However,

values of about 1 nm or less are reasonable in view of the evidence from X-ray crystallography and arguments that only a small lever arm movement should occur prior to the main power-stroke in order to optimize force generation[18].

Parameter values 2: differences in free energy minima between cross-bridge states

Next in Table S1, I consider differences in minimum free energy levels between neighboring states in the cross-bridge model in main Fig. 3. Measurements in reference [19] at 10 °C and 20 °C, suggest that a quantity corresponding to the sum $\Delta G_{on}+\Delta G_{Ps}$ is in the range of 1.5 – 2.8 k_BT. The derivation of this range assumes Q10 in the range, 2.7-3.7[19, 20] for the attachment rate constant with negligible temperture sensitivity of the reverse rate constant. After some fine-tuning in previous studies, we use the value 1.7 k_BT for the sum $\Delta G_{on}+\Delta G_{Ps}$ tentatively assuming that ΔG_{on}=0.7 k_BT and ΔG_{Ps} =1 k_BT based on reasoning in [18] which suggests that both these actomyosin binding strengths are attributed mainly to weak electrostatic interactions. Importantly, the uncertainties in the distribution of the total free energy change ($\Delta G_{on}+\Delta G_{Ps}$) between ΔG_{on} and ΔG_{Ps} are likely to be of negligible significance under physiological conditions. This follows because models that merge the two transitions into one give quite similar predictions as models were the transitions are treated separately[17].

The free energy drop, ΔG_{LH}, associated with the main power-stroke transition (from AMD_L to AMD_H) is likely to be in the range 11 - 19 k_BT based on a sum of ΔG_{LH} + ΔG_{HR} in the range 12-20 kBT[6, 7] estimated from power-stroke distances and stiffness measurements from optical tweezers studies of full length myosin. Furthermore, an observed maximum isometric force in optical traps of 17 pN[21] provides a direct estimate of the difference between the free energy minima of the AMD_L and AMD_H states. With a stiffness of 2.8 pN/nm, a force of 17 pN corresponds to a strain of 6.1 nm and an thus an elastic energy of $2.8 \times 6.1^2/2$= 52.1 pN nm ≈ 13 k_BT which can be taken as a direct estimate of ΔG_{LH}. Based on these results, together with thermodynamic efficiency of less than 50 %[8], a quantity x_2-x_3=1 nm and ΔG_{HR} = 2k_BT, we take ΔG_{LH} = 14 k_BT in our simulations. The value ΔG_{HR} = 2k_BT relies on a value >1.6 k_BT for free-energy differences betweeen the two actomyosin-ADP states found in solution studies[22]. Furthermore, a cross-bridge stiffness as 2.8 pN/nm and a quantity x_2-x_3=2.5 nm (the highest literature value) suggest a free-energy drop ΔG_{HR} in connection with the second power-stroke of 2.1 k_BT. In view of these results we use ΔG_{HR} = 2 k_BT in the simulations. This value also turns out to be of correct magnitude to account for the high-force deviation of the force-velocity relationship and the molecular effects of the small molecular compound amrinone[16].

The free energy change associated with the actual Pi-release step (Table S1) is 3.1 k_BT, assuming an intracellular Pi-concentration of 0.5 mM. Further, the free energy change associated with the ATP hydrolysis/recovery stroke is k_BT ln(10)≈2.3 k_BT, based on the equilibrium constant K_3=10 (Table S2). Now, adding these values (2.3+3.1) k_BT =5.4 k_BT to the sum ($\Delta G_{on}+\Delta G_{Ps}+\Delta G_{LH}+\Delta G_{HR}$) = 17.7 k_BT we arrive at a total sum of 23.1 k_BT leaving approximately a 2 k_BT drop in free energy to be associated with ATP induced actomyosin dissociation to add up to 25 k_BT drop in free energy upon turnover of one ATP molecule. Such a low drop in free energy in the latter step is consistent with a high thermodynamic efficiency (cf. [23]).

Parameter values 3: rate constants

Starting by considering the rate constants for the ATP hydrolysis (Table S2) on the active site of myosin lumped together with the recovery stroke, litterature data suggest values for the sum of the forward and reverse transitions (k_{+3}+k_{-3}) in the range 300-500 s^{-1} with an equilibrium constant[24] K_3 of 4-10. These values are based on solution kinetics results at lower temperature[24, 25] so they also rely on the assumption of a Q_{10} value in the range 3-4 from other studies[24]. What is actually used in the simulations is k_{+3}+k_{-3} = 220 $^{-1}$ and K_3 =10. The low value of k_{+3}+k_{-3} used, reflects a

previously used range of temperatures from 25-30 °C. For the purpose of the present work $k_{+3}+k_{-3}$ has been kept at its previous value[5, 17, 26-28]. However, for future work it should be increased about two-fold to the proper range which actually would be expected to further increase the model power-output slightly.

Now, assuming k_{+3}=400 s^{-1} (as appropriate for 30 °C according to above discussion) and that Q_{10} is 2.8-5[20, 24] for the maximum actomyosin ATP turover rate (V_{max}) in solution, then measurements of V_{max} at 20-25 °C giving values in the range 13-50 s^{-1} [20, 24] suggest that the parameter k_{on}', determining the rate constant of cross-bridge attachment, would be in the range 40-150 s^{-1}. This range would be broader (50-240 s^{-1}) if k_{+3}=200 s^{-1} as actually assumed here. In the simulations, I thus set k_{on}' = 130 s^{-1}, approximately in the middle of the latter range.

The constant k_{P+}', that is a main determinant of the transition rate from the pre-power-stroke state (AMD_{PP}) to the phosphate release state (AMD_{PiR}) (cf. main Fig. 3), was introduced[3], based on previous structural evidence[18] in modelling to account for the effects of the small molecular myosin inhibitor blebbistatin. The exact numerical value of k_{P+}' is unknown. However, the previous work[3] suggests that the numerical value is sufficiently high to have limited effect on the ATP turnover rate and on the maximum sliding velocity under physiological conditions (in the absence of drugs). Whereas k_{P+}' = 1000 s^{-1} was initially used, an increase of k_{P+}' from 1000 s^{-1} to 3000 s^{-1} was implemented more recently[17] because it dramatically increases the maximum velocity (30 %) with minimal (<3 %) effects on maximum isometric tension, rate of rise of isometric force, steady-state ATP turnover rate and shape (curvature; e.g. maximum power) of the force-velocity relationship. Such an increase in velocity was found essential when other minor changes (in x_1 and x_{11}) were implemented to account for effects of varied [Pi] on the force-velocity relationship.

The rate constant of cross-bridge detachment at the end of the power-stroke and in the drag-stroke region[3, 26, 29] is the primary determinant of the maximal shortening velocity although this is also modulated by the value of k_{P+}' and the degree of linearity of the cross-bridge stiffness as outlined above and in the main paper.

The cross-bridge detachment, with transition from the AMD_H state to the MT state in Fig. 3, is governed by a rate constant k_{diss}:

$$k_{diss}(x) = \frac{k_5(x)k_{off}(x)}{(k_5(x)+k_{off}(x))} \qquad (1)$$

where

$$k_{off}(x)= \frac{k_2(x)k_6[MgATP]}{\frac{k_6}{K_1}+(k_2(x)+k_6)[MgATP]} = \frac{k_2(x)[MgATP]}{\frac{1}{K_1}+\frac{k_2(x)}{k_6}[MgATP]+[MgATP]} \qquad (2)$$

This means that k_6, K_1 as well as the constants determining the numerical values of $k_5(x)$ (primarily k_{-5}) and $k_2(x)$ (primarily k_2 and x_{crit}) will have major role in determining the maximum velocity of shortening while also having effects on the shape of the force-velocity relationship. Fortunately, quantiative estimates of k_6, k_2 and K_1 can be found in the litterature[30] for fast rabbit myosin at ionic strengths in the range 100 – 200 mM (as used here) and temperature of 25 °C. I directly use the values of k_6 and K_1 from that paper because they exhibited limited temperature dependence. However, the value of k_2 increased with temperature which I take into account here in setting k_2=2000 s^{-1} in the simulations. From the values of k_6, k_2, and K_1 from[30] it follows that k_{-5} must have a similar magnitude as k_2 and k_6 to account for the high maximum velocity of shortening. However, at temperatures >25 °C both k_2

and rate constants associated with ADP release are likely to be of similar magnitude[30]. Here, I therefore set $k_{-5} = 2000\ s^{-1}$, similar to the value of k_2.

The parameter x_{crit}, which defines the strain-dependence of the rate function $k_2(x)$ had to be taken as 0.6 nm to account for the observed maximum velocity of shortening in the range 13000-18000 nm per half-sarcomere per second for fast mammalian muscle at 30 ^oC (see [26] and references therein) with other parameter values as in Tables S1-S2. This was necessary, despite the fact that ultrafast force clamp records using fast myosin subfragment 1 from the mouse at 20 ^oC suggest that $x_{crit}<0.2$ nm. The discrepancy could have different causes. One possibility is that the experimental data from mouse subfragment 1 at 20 ^oC do not reflect the properties of full length rabbit psoas muscle at 30 ^oC. Another possibility is that the cross-bridge elasticity is non-linear also in muscle as found previously for isolated myosin molecules[7]. In the latter case, the high maximum velocity of shortening of mammalian muscle is explained without any strain dependence of $k_2(x)$, i.e. with $x_{crit}=0$ nm.

Table S1. Parameter values[a] determining free energy profiles (main Fig. 3B) for simulation of contractile properties of fast mammalian muscle at 30 °C

Parameter	Numerical value used	Range from the litterature	Comments
x-values for positions of free energy minima of different states			
x_1 ($AMDP_{PP}$)	7.2 nm	Slightly higher than x_{11} [18]	
x_{11} ($AMDP_{PiR}$ and AMD_L)	6.7 nm	7-13 nm[6]	See Supporting Text
x_2 (AMD_H)	1.0 nm	0.9-2.5 nm[14, 15]	Data from myosin subfragment 1 of fast mouse muscle; 22 °C; ionic strength < 30-50 mM[14] or from rabbit full length myosin at 20 °C[7]
x_3 (AM/AMD)	0 nm	0 nm	By definition
Differences in free energies between free energy minima of neighboring states			
$\Delta G_{AMDP\text{-}AMDP} \equiv \Delta G_{on}$ (AMDP-$AMDP_{PP}$)	0.7 k_BT	Based on $\Delta G_{on} + \Delta G_{Ps}$ in the range 1.5-2.8 k_BT[19, 20] and reasoning in [18].	Both parameters are expected to be ionic strength dependent and rather weak but stereospecific.
$\Delta G_{AM'DPpp\text{-}AMDPpir} \equiv \Delta \mathbf{G_{Ps}}$ ($AMDP_{PP}$ – $AMDP_{PiR}$)	1 k_BT		
$\Delta G_{AM'DPpir\text{-}AMDL} \equiv \Delta \mathbf{G_P}$ ($AMDP_{Pir}$ – AMD_L)	$k_BT \ln([Pi]/K_C)$ ≈3 k_BT at 0.5 mM Pi	See parameter K_C below	
$\Delta G_{AMDL\text{-}AM'DH} \equiv \Delta G_{LH}$ (AMD_L- AMD_H)	14 k_BT	11-19 k_BT[6, 7 21]	
$\Delta G_{AMDH\text{-}AM/AMD} \equiv \Delta G_{HR}$ (AMD_H – AMD/AM)	2 k_BT	1.6 – 2.1 k_BT[16 22]	
ΔG_{ATP}	13.1 + ln ([MgATP]/ ([MgADP][Pi]) k_BT	~25 k_BT at cellular substrate and product concentrations[8, 31]	
Cross-bridge stiffness			
ks (strongly bounds states)	2.8 pN/nm	1.5-3 pN/nm[6, 7, 10]	

Footnotes to Table S1

[a] The parameter values were from myosin motor fragments (subfragment 1, heavy meromyosin or full length myosin) from fast skeletal muscle of rabbit at 30°C, ionic strength 100-200 mM, pH 7-8 unless otherwise stated. Detailed discussion of the origin of the parameter values in the Supporting Text.

Table S2. Parameter values[a] defining rate functions and kinetic constants in main Fig. 3 for simulation of contractile properties of fast mammalian muscle at 30 °C.

Parameter	Numerical value used	Range from the litterature	Comments
$k_{+3} + k_{-3}$	220 s^{-1}	300-500 s^{-1} ([24, 25, 32]) assuming Q10 in range 3-4 [24]	
K_3	10	2-10[24, 25, 32]	
k_{on}'	130 s^{-1}	50 – 240 s^{-1} [20, 24]	
k_{P+}'	3000 s^{-1}	Initially[3] taken as 1000 s^{-1} but to minimize inhibition of sliding velocity later increased to 3000 s^{-1} ([17])	
k_{-5}	2000 s^{-1}	>2000 $^{-1}$	See Supporting Text.
K_C	10 mM	1 – 10 mM[19]	From fast skinned muscle fiber phosphate transients, from data at 20-25 °C
x_{crit}	0.6 nm	< 0.2 nm[33]	See Supporting Text
k_6	5000 s^{-1}	>3500 s^{-1} [30]	
Physiological [Pi]	0.5 mM	~ 0.5 mM [34]	
Physiological [MgATP]	5 mM	5-10 mM[35 36]	In resting muscle
K_1	1.7 mM^{-1}	~1.7 mM^{-1} [34]	
k_2	2000 s^{-1}	1500 – 2000 s^{-1} [34]	Temperature corrected (Q_{10}=2.3) from 25 °C

Footnotes to Table S2

[a] The parameter values were from myosin motor fragments (subfragment 1, heavy meromyosin or full length myosin) from fast skeletal muscle of rabbit at 30°C, ionic strength 100-200 mM, pH 7-8 unless otherwise stated.

Table S3. Variation with x of rate functions used for simulations in main paper

Rate	Expression
$k_{on}(x)$	k_{on}' exp(Δ G_{on} - ks $(x-x_1)^2$/ k_BT + ksw$(x-x_1)^2$/ k_BT)
Reversal of $k_{on}(x)$	k_{on}' exp(ks $(x-x_1)^2/k_BT$ ksw$(x-x_1)^2/k_BT$)
$k_{Pr}(x)$	k_{P+}'exp(Δ $G_{AMDPpp-AMDPpir}$/2 -(ks/2)$(x-x_{11})^2$/ $(2k_BT)$ +(ks/2)$(x-x_1)^2/(2k_BT)$))
Reversal of $k_{Pr}(x)$	k_{P+}'[Pi]/([Pi]+Kp) exp (Δ $G_{AMDPpp-AMDPpir}$ /2+(ks/2)$(x-x_1)^2/(2k_BT)$-(ks/2)$(x-x_w)^2/(2k_BT)$)
$K_{LH}(x)$	$K_{LH}(x)=k_{LH+}(x)/k_{LH-}(x)$
$k_{LH+}(x)$	$k_{LH-}(x)$ exp(Δ $G_{AMDL-AMDH}$ +(ks/2)$(x-x_{11})^2/(k_BT)$-(ks/2)$(x-x_2)^2/(k_BT)$)
$k_{LH-}(x)$	6000 s^{-1}
Kc	10 mM
$k_5(x)$	k_{-5} exp(Δ G_{HR} +(ks/2)$(x-x_2)^2/(k_BT)$-(ks/2)$(x-x_3)^2/(k_BT)$)
$k_2(x)$	$k_2 \exp\left(\frac{k_s \cdot \lvert x - x_3 \rvert \cdot x_{crit}}{k_B T}\right)$

Footnotes to Table S3

[a] For further definition of symbols, see main Fig. 3 and for parameter values used, see Tables S1 and S2. More details in [3].

Supporting References

1. Hill, T.L. Theoretical formalism for the sliding filament model of contraction of striated muscle. Part I. *Prog. Biophys. Mol. Biol.* **28**, 267-340 (1974).
2. Huxley, A.F. Muscle structure and theories of contraction. *Prog Biophys Biophys Chem* **7**, 255-318 (1957).
3. Rahman, M.A., Usaj, M., Rassier, D.E. & Mansson, A. Blebbistatin Effects Expose Hidden Secrets in the Force-Generating Cycle of Actin and Myosin. *Biophys. J.* **115**, 386-397 (2018).
4. Gillespie, D.T. A general method for numerically simulating the stochastic time evolution of coupled chemical reactions. *J Comp Phys* **22**, 403-434 (1976).
5. Månsson, A. Actomyosin based contraction: one mechanokinetic model from single molecules to muscle? *J. Muscle Res. Cell Motil.* **37**, 181-194 (2016).
6. Månsson, A., Usaj, M., Moretto, L. & Rassier, D.E. Do Actomyosin Single-Molecule Mechanics Data Predict Mechanics of Contracting Muscle? *Int J Mol Sci* **19** (2018).
7. Kaya, M. & Higuchi, H. Nonlinear elasticity and an 8-nm working stroke of single myosin molecules in myofilaments. *Science* **329**, 686-689 (2010).
8. Barclay, C.J., Woledge, R.C. & Curtin, N.A. Inferring crossbridge properties from skeletal muscle energetics. *Prog. Biophys. Mol. Biol.* **102**, 53-71 (2010).
9. Lewalle, A., Steffen, W., Stevenson, O., Ouyang, Z. & Sleep, J. Single-molecule measurement of the stiffness of the rigor myosin head. *Biophys. J.* **94**, 2160-2169 (2008).
10. Linari, M., Caremani, M., Piperio, C., Brandt, P. & Lombardi, V. Stiffness and fraction of Myosin motors responsible for active force in permeabilized muscle fibers from rabbit psoas. *Biophys. J.* **92**, 2476-2490 (2007).
11. Caremani, M., Melli, L., Dolfi, M., Lombardi, V. & Linari, M. The working stroke of the myosin II motor in muscle is not tightly coupled to release of orthophosphate from its active site. *J Physiol* **591**, 5187-5205 (2013).
12. Smith, D.A. A new mechanokinetic model for muscle contraction, where force and movement are triggered by phosphate release. *J. Muscle Res. Cell Motil.* **35**, 295-306 (2014).
13. Offer, G. & Ranatunga, K.W. A cross-bridge cycle with two tension-generating steps simulates skeletal muscle mechanics. *Biophys. J.* **105**, 928-940 (2013).
14. Capitanio, M. et al. Two independent mechanical events in the interaction cycle of skeletal muscle myosin with actin. *Proc. Natl. Acad. Sci. U. S. A.* **103**, 87-92 (2006).
15. Kaya, M., Tani, Y., Washio, T., Hisada, T. & Higuchi, H. Coordinated force generation of skeletal myosins in myofilaments through motor coupling. *Nature communications* **8**, 16036 (2017).
16. Albet-Torres, N. et al. Drug effect unveils inter-head cooperativity and strain-dependent ADP release in fast skeletal actomyosin. *J. Biol. Chem.* **284**, 22926–22937 (2009).
17. Mansson, A. The effects of inorganic phosphate on muscle force development and energetics: challenges in modelling related to experimental uncertainties. *J. Muscle Res. Cell Motil.* (2019).
18. Llinas, P. et al. How actin initiates the motor activity of Myosin. *Dev Cell* **33**, 401-412 (2015).
19. Dantzig, J.A., Goldman, Y.E., Millar, N.C., Lacktis, J. & Homsher, E. Reversal of the cross-bridge force-generating transition by photogeneration of phosphate in rabbit psoas muscle fibres. *J Physiol* **451**, 247-278 (1992).
20. Brenner, B. & Eisenberg, E. Rate of force generation in muscle: correlation with actomyosin ATPase activity in solution. *Proc. Natl. Acad. Sci. U. S. A.* **83**, 3542-3546 (1986).
21. Takagi, Y., Homsher, E.E., Goldman, Y.E. & Shuman, H. Force generation in single conventional actomyosin complexes under high dynamic load. *Biophys. J.* **90**, 1295-1307 (2006).

22. Sleep, J.A. & Hutton, R.L. Exchange between inorganic phosphate and adenosine 5'-triphosphate in the medium by actomyosin subfragment 1. *Biochemistry.* **19**, 1276-1283 (1980).
23. Eisenberg, E. & Greene, L.E. The relation of muscle biochemistry to muscle physiology. *Annu. Rev. Physiol.* **42**, 293-309 (1980).
24. Woledge, R.C., Curtin, N.A. & Homsher, E. Energetic aspects of muscle contraction. (Academic Press, London; 1985).
25. Johnson, K.A. & Taylor, E.W. Intermediate states of subfragment 1 and actosubfragment 1 ATPase: reevaluation of the mechanism. *Biochemistry (Mosc).* **17**, 3432-3442. (1978).
26. Månsson, A., Persson, M., Shalabi, N. & Rassier, D.E. Non-linear actomyosin elasticity in muscle? *Biophys. J.* **116**, 330–346 (2019).
27. Månsson, A. Comparing models with one versus multiple myosin-binding sites per actin target zone: The power of simplicity. *J. Gen. Physiol.* **151**, 578-592 (2019).
28. Rahman, M.A., Salhotra, A. & Månsson, A. Comparative analysis of widely used methods to remove nonfunctional myosin heads for the in vitro motility assay. *J. Muscle Res. Cell Motil.* **39**, 175-187 (2018).
29. Persson, M., Bengtsson, E., ten Siethoff, L. & Månsson, A. Nonlinear cross-bridge elasticity and post-power-stroke events in fast skeletal muscle actomyosin. *Biophys. J.* **105**, 1871-1881 (2013).
30. Nyitrai, M. et al. What limits the velocity of fast-skeletal muscle contraction in mammals? *J. Mol. Biol.* **355**, 432-442 (2006).
31. Pate, E. & Cooke, R. A model of crossbridge action: the effects of ATP, ADP and Pi. *J. Muscle Res. Cell Motil.* **10**, 181-196. (1989).
32. Sleep, J., Irving, M. & Burton, K. The ATP hydrolysis and phosphate release steps control the time course of force development in rabbit skeletal muscle. *J Physiol* **563**, 671-687 (2005).
33. Capitanio, M. et al. Ultrafast force-clamp spectroscopy of single molecules reveals load dependence of myosin working stroke. *Nature methods* **9**, 1013-1019 (2012).
34. Debold, E.P., Turner, M.A., Stout, J.C. & Walcott, S. Phosphate enhances myosin-powered actin filament velocity under acidic conditions in a motility assay. *Am J Physiol Regul Integr Comp Physiol* **300**, R1401-1408 (2011).
35. Kuno, S.-y. & Itai, Y. Muscle Energetics during Exercise by ³¹P NMR. *The Annals of physiological anthropology* **11**, 313-318 (1992).
36. Kushmerick, M.J., Moerland, T.S. & Wiseman, R.W. Mammalian skeletal muscle fibers distinguished by contents of phosphocreatine, ATP, and Pi. *Proc. Natl. Acad. Sci. U. S. A.* **89**, 7521-7525 (1992).
